# Quantitative analysis and virulence phenotypes of atypical enteropathogenic *Escherichia coli* (EPEC) acquired from diarrheal stool samples from a Midwest US hospital

**DOI:** 10.1080/19490976.2020.1824562

**Published:** 2020-11-01

**Authors:** MJ Carlino, SE Kralicek, SA Santiago, LM Sitaraman, AT Harrington, Gail A. Hecht

**Affiliations:** a Department of Microbiology and Immunology, Loyola University Chicago, Maywood, IL, USA; b Department of Medicine, Division of Gastroenterology and Nutrition, Loyola University Chicago, Maywood, IL, USA; c Stritch School of Medicine, Loyola University Chicago, Maywood, IL, USA; d Department of Pathology and Laboratory Medicine, Loyola University Chicago, Maywood, IL, USA; e Department of Medical Service, Edward Hines Jr. VA Hospital, Hines, IL

**Keywords:** BioFire gastrointestinal panel, atypical EPEC, actin pedestal, bacterial adherence, transepithelial electrical resistance

## Abstract

Infectious diarrhea causes approximately 179 million illnesses annually in the US. Multiplex PCR assays for enteric pathogens detect enteropathogenic *Escherichia coli* (EPEC) in 12–29% of diarrheal stool samples from all age groups in developed nations. The aim of this study was to isolate and characterize EPEC from diarrhea samples identified as EPEC positive by BioFire Gastrointestinal Panel (GIP). EPEC is the second most common GIP-detected pathogen, equally present in sole and mixed infections peaking during summer months. EPEC bacterial load is higher in samples with additional pathogens. EPEC-GIP-positive stool samples were cultured on MacConkey II agar and analyzed by colony PCR for *eaeA* and *bfpA* to identify and classify EPEC isolates as typical (tEPEC) or atypical (aEPEC). EPEC were not recovered from the majority of stool samples with only 61 isolates obtained from 277 samples; most were aEPEC from adults. *bfpA*-mRNA was severely diminished in 3 of 4 *bfpA*-positive isolates. HeLa and SKCO-15 epithelial cells were infected with EPEC isolates and virulence-associated phenotypes, including adherence pattern, attachment level, pedestal formation, and tight junction disruption, were assessed. All aEPEC adherence patterns were represented with diffuse adherence predominating. Attachment rates of isolates adhering with defined adherence patterns were higher than tEPEC lacking *bfpA* (Δ*bfpA*). The majority of isolates formpedestals. All but one isolate initially increases but ultimately decreases transepithelial electrical resistance of SKCO-15 monolayers, similar to Δ*bfpA*. Most isolates severely disrupt occludin; ZO-1 disruption is variable. Most aEPEC isolates induce more robust virulence-phenotypes *in vitro* than Δ*bfpA*, but less than tEPEC-E2348/69.

## Introduction

Enteropathogenic *Escherichia coli* (EPEC) arediarrheagenic pathogens that induce characteristic attaching and effacing (A/E) lesions on the surface of host enterocytes. EPEC are sub-classified as typical (tEPEC) or atypical EPEC (aEPEC) by the respective presence or absence of the *E. coli* adherence factor plasmid (pEAF), which harbors the gene encoding the major pilin (*bfpA*) of the bundle-forming pilus (BFP). BFP is a type IV pilus that mediates bacteria–bacteria interactions, promoting attachment of dense clusters of bacteria to host intestinal epithelial cells.^1^ These clusters are referred to as microcolonies that characterize the attachment pattern known as localized adherence (LA).^2^ BFP is also important for bringing tEPEC and host cells into close proximity via BFP retraction, thus increasing delivery of bacterial effector proteins into host cells.^3^ In the absence of *bfpA*, aEPEC are unable to form the LA pattern and virulence is highly variable.^4^

Infectious diarrhea results from disruption of the homeostatic balance of ion and water transport in the gastrointestinal tract.^5^ Enteric pathogens can modulate ion or water transport directly by producing toxins or effector proteins, or by inducing inflammatory responses that perturb the normal function of intestinal epithelia.^6^ In the US, there are estimated 179 million cases of acute infectious diarrhea per year that result in 228,744 hospitalizations and 2,612 deaths.^7^ tEPEC cause infantile diarrhea in developing nations. However, the presence of EPEC in developed countries and in adults has been underestimated due to the lack of rapid and routinely used screening tools. In fact, two meta-analyses of the prevalence of gastrointestinal pathogens (1980–2008) revealed that EPEC incidence in developed nations is severely underreported or not reported compared to other pathogens, especially in studies of adults. These studies state that EPEC are detected in only 0.1–1.3% of the diarrheal samples from adults in developed nations.^8,9^ A US-specific study from 2002 to 2004 found EPEC in 4.3% of samples.^10^ In contrast, recent epidemiologic investigations aided by multiplex PCR analysis found EPEC to be present in 12–29% of diarrheal stool samples across all age groups in developed and developing nations alike.^11^ Specifically in the US, a multicenter evaluation of the BioFire FilmArray Gastrointestinal Panel (GIP) (BioFire Diagnostics), a multiplex PCR assay that screens diarrheal samples for 22 enteric pathogens, similarly showed EPEC to be the most prevalent pathogen detected, present in 22% of samples across all age groups.^12^ BioFire GIP was approved for use in the US in 2014, and is now used in many clinical microbiology labs. Although data regarding aEPEC prevalence in the US is lacking, studies in Australia, UK, and England detected aEPEC in 95–99% of EPEC-positive samples, with similar reports from developing countries indicating aEPEC occurrence has emerged over tEPEC.^11^

aEPEC pathogenicity, however, is controversial. aEPEC can cause severe diarrhea but is also found in ~3-20% of asymptomatic individuals and sometimes with co-infecting agents.^10,11,13^ In addition, an aEPEC strain causing an infantile diarrheal outbreak was given to 15 college student volunteers with mean age of 24. Interestingly, none developed diarrhea, despite subsequent positive stool cultures for the organism.^14^ Other human volunteer studies, however, reported that ingestion of tEPEC caused diarrhea in 85–90% of individuals compared to only 12.5–22% of those infected with tEPEC-E2348/69 lacking *bfpA* (*ΔbfpA*) or cured of pEAF.^15,16^ The response to ingestion of a clinical aEPEC strain was intermediate, causing diarrhea in 55% of volunteers indicating that this aEPEC strain harbors additional virulence factors compared to Δ*bfpA*.^16^ aEPEC have also been associated with diarrheal outbreaks of children and adults in Japan, Finland, the US, and China.^17–23^

aEPEC pathogenic mechanisms are assumed to be similar to tEPEC due to the presence of the locus of enterocyte effacement (LEE) pathogenicity island, however, this is largely unsubstantiated. One factor contributing to differences between tEPEC and aEPEC is the lack of BFP in aEPEC, as BFP is not only involved in host and bacterial adherence, but also in efficient effector delivery and tight junction disruption.^1–3^ aEPEC often house virulence factors not traditionally ascribed to tEPEC and represent a large heterogeneous group of organisms.^24–26^ Such virulence factors and genetic diversity likely help compensate for the lack of BFP and account for the variation in pathogenicity among different aEPEC strains. Thus, this study investigated the ability of individual aEPEC strains to induce virulence-associated phenotypes.

In order to better understand the pathogenicity of aEPEC strains from a developed nation, this study assessed *in vitro* virulence-associated phenotypes induced by aEPEC isolated from human diarrheal samples obtained from a US Midwest hospital and compared them to those induced by wildtype tEPEC-E2348/69 and Δ*bfpA*. Quantitative EPEC load in stool samples, adherence pattern, attachment level, actin pedestal formation, and disruption of tight junction (TJ) structure and function are reported herein.

## Results

### EPEC is the second most GIP-detected enteric pathogen

**Figure 1.**
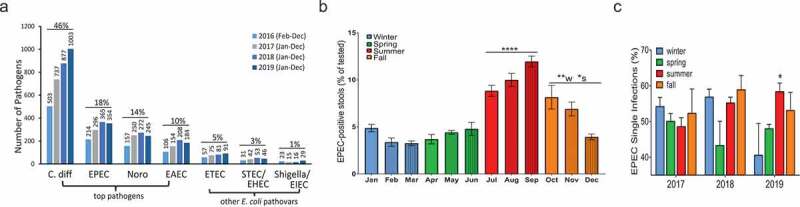
BioFire Gastrointestinal Panel (GIP) data from LUMC. (a) Number of diarrheal specimens collected between Feb 2016 and Dec 2019 that are positive for the indicated enteric pathogen. Top pathogens, *C. difficile* (C. diff), EPEC, Norovirus (Noro), and enteroaggregative *E. coli* (EAEC), and other *E. coli* pathovars, enterotoxigenic *E. coli* (ETEC), Shiga-toxigenic/enterohemorrhagic *E. coli* (STEC/EHEC), and Shigella/enteroinvasive *E. coli* (EIEC), are reported. Percentage of positive samples is indicated for each pathogen. EPEC is the 2^nd^ most detected pathogen after *C. difficile* and is more prevalent than all other *E. coli* pathovars combined. (b) Average percentage of EPEC-positive samples compared to number of samples tested per month from Feb 2016-Dec 2019. EPEC infection peaks in summer months July-Sept and remains high in fall months Oct-Nov. 1-way ANOVA performed with Tukey post-hoc test. **** *p* < .0001 compared to all other months; ** *p* < .01 compared to winter months, and * *p* < .05 compared to spring months. (c) EPEC co-infection status with other enteric pathogens was examined from Jan 2017-Dec 2019. Percentage of EPEC single infections is displayed with similar averages per year (51.72 ± 1.04%), however, in 2019 EPEC single infections are significantly higher in summer months compared to those in winter months. 1-way ANOVA performed with Tukey post-hoc test; * *p* < .05

Loyola University Medical Center (LUMC) Clinical Microbiology Laboratory acquired GIP in early 2016. GIP testing at LUMC is performed for persons presenting with diarrhea at the discretion of the ordering physician or nurse with orders placed at LUMC urgent care centers, emergency department, inpatient, or by the primary care physician then centrally analyzed by the LUMC Clinical Microbiology Laboratory. Analysis of LUMC GIP data reveals EPEC to be the second most frequently detected pathogen in tested diarrheal stool samples. GIP samples are deemed EPEC positive when the intimin-encoding *eaeA* gene is detected in the absence of Shiga toxin genes, *stx1/stx2*. EPEC represents 18.3% (1229/6734) of positive specimens, second only to *C. difficile* (Figure 1(a)). EPEC are detected more often than all other *E. coli* pathovars combined (Figure 1(a)). GIP does not screen for *bfpA*, therefore, EPEC are not classified as typical or atypical by this assay. EPEC peaks to the highest levels in July and August, begins to decline in October and November with the least number of cases detected December–June (Figure 1(b)). Sole infections of EPEC (single) and those with co-infecting pathogens detected (mixed) are equally represented over 3 years with similar yearly averages of single infections being 51.4 ± 1.8%, 53.7 ± 2.5%, and 50.1 ± 3.0%, for 2017, 2018, and 2019, respectively. However in 2019, sole infections of EPEC peaked in the summer months and were significantly higher than those in winter months (Figure 1(c)). These data indicate that EPEC infection occurs as both a single pathogen, especially during the summer months, but also in the presence of other enteric pathogens.

### Higher EPEC loads are more frequently detected in samples with mixed infection

Higher bacterial loads of EPEC in stool samples are associated with diarrheal symptoms.^27^ Therefore, we quantified EPEC bacterial load using quantitative PCR (qPCR) in 60 randomly collected GIP-EPEC-positive samples received from the LUMC Clinical Microbiology Laboratory between January and April 2019. The consistency and amount of stool submitted in Cary-Blair transport media varied greatly between samples. Consequently, stool input into the DNA extraction and the qPCR analysis varied between samples. To normalize results, a universal bacterial 16S rDNA primer/probe set was used during the qPCR analysis to measure total bacterial load in each sample. This technique has been reported by others to reliably determine total bacterial load from non-uniform samples such as dental cavities and rectal swabs.^28,29^ Relative abundance has also been reported to be associated with symptomatic individuals in the context of *C. difficile*.^30^ Total bacterial 16S rDNA detection also served as an internal control for DNA extraction and amplification. To quantify EPEC load, a newly designed probe (Supplemental Table 1) and *eaeA* primers were used as described.^31^

Validation of the *eaeA* and universal primer/probe sets was performed on tEPEC-E2348/69 genomic DNA extracted using the Qiagen DNeasy Blood and Tissue Kit. The stated limit of detection (LOD) for EPEC by GIP is 200 bacteria, or 1.088 pg DNA assuming 5.44 fg DNA/bacterium.^29,32^ Standard curves ranging from 0.138 to 1.38 × 10^5^ pg DNA were successfully generated for universal bacteria and *eaeA* with R^2^ = 0.998 and efficiency = 1.00 (Supplemental Figure 1). These data indicate the efficiency of the *eaeA-* and universal-primer/probe sets permit quantification with sensitivity similar to GIP.

We also validated the qPCR efficiency to detect known CFUs of tEPEC-E2348/69. tEPEC-E2348/69 overnight culture was serially diluted and enumerated after incubation on Luria-Bertani plates. The remainder of culture was subjected to genomic DNA extraction using the QIAamp PowerFecal DNA Kit and qPCR analysis with *eaeA*- and universal-primer/probes. The efficiency of both qPCR reactions was high, generating standard curves with R^2^ > 0.98 (Supplemental Figure 2). However, the overall sensitivity of the assay was limited to an input of 1.91 × 10^3^ bacteria, nearly ten times higher than the GIP reported LOD (Supplementary Figure 2).^32^

**Figure 2.**
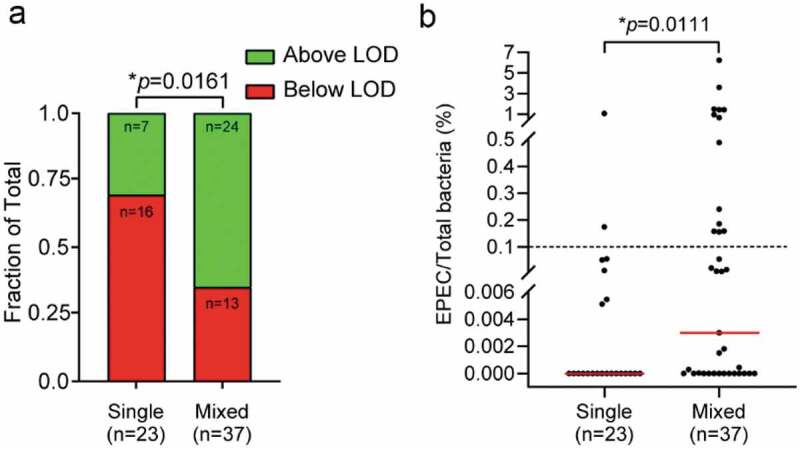
EPEC load is greater in samples with co-infecting pathogens. (a) Proportion of GIP-EPEC-positive samples that are above or below the limit of detection (LOD) of the qPCR assay. Fisher’s exact test for difference between proportions was performed. A greater proportion of samples are above the LOD when EPEC is part of a co-infection (mixed) versus the sole pathogen detected (single). (b) EPEC relative load is displayed as EPEC quantity as percentage of total bacteria with medians (red line) plotted for single (median = 0%) and mixed (median = 2.99x10^−3^%) infections. In most samples (75% – 45/60), EPEC represent <0.1% (dashed line) of total bacteria. EPEC loads tend be higher in mixed infections based on Mann-Whitney U test of rank sums comparing all samples below and above the LOD

Having validated the qPCR assay, EPEC and total bacterial loads were determined for the 60 random GIP-EPEC positive samples collected between January–April, 2019. A standard curve of tEPEC-E2348/69 genomic DNA amplified with each primer/probe set was generated in triplicate for each qPCR run to quantify EPEC and total bacteria. EPEC signal was below the LOD in nearly half (29/60) of the samples, highlighting the extreme sensitivity of GIP detection (Figure 2(a)). To determine if co-infection status influences EPEC bacterial load, the proportion of samples above and below the LOD in each group was compared. EPEC load was more frequently above the LOD in samples harboring additional enteric pathogens as compared to those that only tested positive for EPEC (Figure 2(a)).

To determine the relative load of EPEC in each sample, the quantified values of EPEC were divided by total bacteria and loads compared between samples with single and mixed infections (Figure 2(b)). EPEC represented 0.005% to 1.1% of total bacteria in samples with EPEC as the sole pathogen (Figure 2(b-single)). However, EPEC load was below 0.1% of total bacteria, with a median value of zero, in most of the samples with EPEC as the sole pathogen (Figure 2(b-single)). In contrast, when present with other pathogens, EPEC represented 0.00004% to 6.22% of total bacteria in detected loads (Figure 2(b-mixed)). More than half of the mixed infection samples had EPEC loads greater than 0.1% of total bacteria with a median of 0.00299%, significantly higher than EPEC loads in samples of sole infections (Figure 2(b-mixed)). These data show that EPEC loads are generally higher when EPEC is detected as part of a co-infection compared to when EPEC is the sole pathogen (Figure 2(b)).

### Majority of clinical EPEC isolates are atypical EPEC from adults

**Figure 3.**
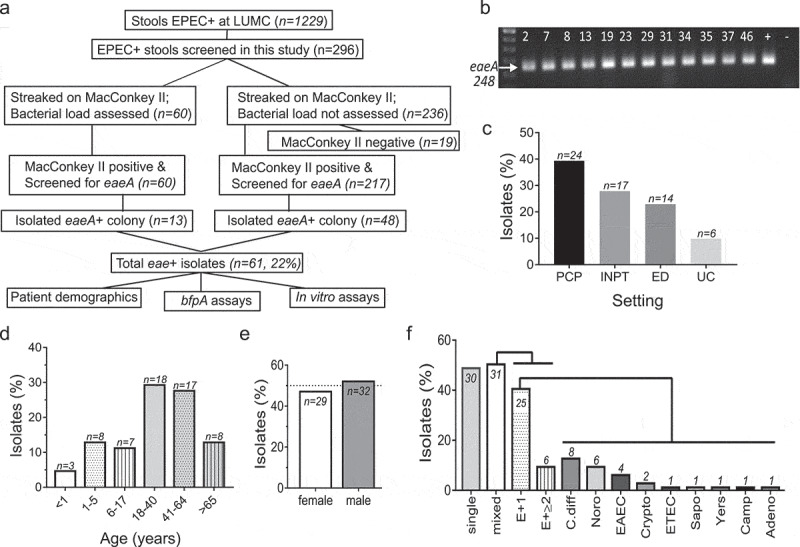
Majority of EPEC isolates represent cases from adult patients and are equally distributed between EPEC as sole pathogen and from mixed infections. (a) Flow chart of the screening process for EPEC-positive diarrheal specimens obtained from the LUMC clinical microbiology laboratory between 2016–2019. GIP identified EPEC in 1,229 stool specimens at LUMC of which 296 were received for this study. All 296 stools were subjected to aerobic growth on MacConkey II agar. Of these, 60 random samples were subjected to qPCR analysis to detect EPEC bacterial load. Colonies from MacConkey II plates (277/296) were screened for *eaeA* via colony PCR resulting in 61 purified EPEC isolates. For the 61 purified EPEC isolates, patient demographics were collected, and *bfpA* screening and *in vitro* assays were performed. (b) Representative image of PCR products of individual colonies from diarrheal specimens probed for *eaeA-*248 bp amplicon. (c-f) A retrospective chart review was conducted to obtain basic patient demographic information. (c) Proportion of clinical isolates from the LUMC setting where GIP was ordered by a physician or nurse; primary care physician (PCP), inpatient (INPT), emergency department (ED), or urgent care (UC). (d) Proportion of clinical EPEC isolates by age group with “n” displayed for each age group. Greatest number of isolates are from patients 18–64 years old (35/61; 57.3%). (e) An equal proportion of male/female patients are represented in clinical EPEC isolates. (f) Similar proportions of isolates come from samples with EPEC as the sole pathogen (single) and those with co-infecting pathogens (mixed) as determined by GIP. Examination of mixed infections reveals EPEC plus 1 other pathogen (E + 1) occurs more frequently than EPEC plus 2 or more pathogens (E+≥2). *C. difficile* (C. diff) predominates E + 1 infection; other pathogens include Noro, EAEC, Cryptosporidium (Crypto), ETEC, Sapovirus (Sapo), *Yersinia* (Yers), *Campylobacter* (Camp), and Adenovirus (Adeno)

Two hundred ninety-six out of 1229 GIP-EPEC-positive stool samples randomly collected from the LUMC Microbiology Laboratory between 2016 and 2019 were plated on MacConkey II agar. Nineteen samples failed to grow on MacConkey II agar (Figure 3(a)). Individual colonies from the remaining 277 positive MacConkey II growth samples were screened for the *eaeA* gene via colony PCR (Figure 3(a-b)). Colonies positive for *eaeA* were streaked again on MacConkey II agar and rescreened for *eaeA* to verify isolate identity as EPEC. Sixty-one of 277 (22.0%) total samples screened yielded EPEC isolates, identified by amplification of the *eaeA*-248 bp fragment (Figure 3(b)).

Patient demographic data corresponding to the 61 clinical EPEC isolates were collected. The largest number of isolates are from patients with GIP testing ordered by their LUMC primary care physician (PCP) (39% (24/61)) (Figure 3(c)). Isolates from LUMC inpatient and emergency department represent 28% (17/61) and 24% (14/61) of samples, respectively, while the fewest number of isolates are from patients seen in urgent care (UC) settings (10% (6/61)) (Figure 3(c)). 57% (35/61) of isolates are from adults between 18 and 64 years of age (Figure 3(d)). Isolates from patients in age groups 1–5, 6–17, and **≥**65 years are equally distributed at 13.1%, 11.5%, and 13.1%, respectively, while only 3 isolates are from infants under 1 year of age (4.92%) (Figure 3(d)). EPEC isolates are equally distributed between females and males at 47.5% and 52.5%, respectively (Figure 3(e)). Approximately half of the isolates are from samples positive only for EPEC (49.2%) while the remainder (50.8%) identified additional pathogens (Figure 3(f)). Of the isolates from samples detecting multiple pathogens, 80.7% have only one additional pathogen, most commonly *C. difficile*, Norovirus, or enteroaggregative *E. coli* (EAEC). The remainder (19.3%) is positive for 2 or more pathogens (Figure 3(f)).

**Figure 4.**
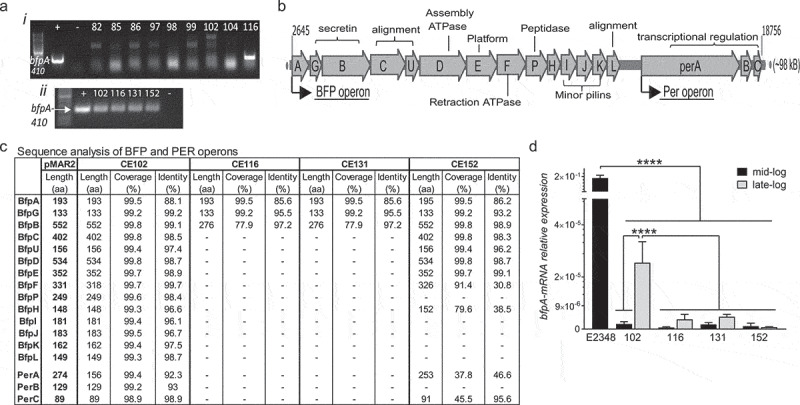
Majority of clinical EPEC isolates lack *bfpA* or have low *bfpA* transcript levels. (a) Representative images of *eaeA*-positive isolates screened for a *bfpA-*410 bp amplicon via PCR on (i) colonies or (ii) purified plasmid preparations. 93.4% (57/61) are aEPEC based on lack of *bfpA* detection and 4 are *bfpA*-positive (CE102, CE116, CE131, and CE152). (b) Schematic of the bundle-forming pili (BFP) operon and the transcriptional regulator PER operon from pMAR2, the EAF plasmid present in tEPEC-E2348/69. (c) Plasmid preparations of *bfpA*-positive isolates were subjected to exonuclease treatment to remove genomic DNA, shotgun sequencing, and *de novo* assembly then compared to pMAR2. Isolate CE102 is most similar to pMAR2 although *perA* is truncated. CE116 and CE131 lack nearly all genes of the BFP operon and the entire PER operon. CE152 is intermediate lacking the peptidase and minor pilin genes, and has major alterations in the PER operon. (d) tEPEC-E2348/69 and the 4 *bfpA*-positive isolates (CE102, CE116, CE131, and CE152) were grown to mid- and late-log phase, RNA extracted, and RT-qPCR performed. Relative *bfpA*-mRNA expression levels (mean±SEM) are displayed and analyzed using 1-way ANOVA. tEPEC-E2348/69 relative *bfpA*-mRNA expression is significantly higher than each of the clinical isolates (**** *p* < .0001). Isolate CE102 *bfpA*-mRNA relative expression in late-log phase is significantly higher compared to itself at mid-log phase and compared to all other isolates at mid- and late-log phase (****p < .0001). Although not statistically significant, isolates CE116, CE131, and CE152 *bfpA*-mRNA relative expression levels tend to be higher in late-log phase over mid-log phase

To determine if the 61 isolates were typical or atypical EPEC, colony PCR was used to detect a 410 bp fragment of *bfpA*. The majority of isolates are aEPEC with 94% (57/61) lacking *bfpA* and only 4/61 isolates being positive for *bfpA* via colony PCR (CE102, CE116, CE131, and CE152) (Figure 4(a-*i*)). PCR of large construct plasmid preparations of these four isolates confirms the presence of *bfpA* (Figure 4(a-*ii*)). Plasmid preparations were treated with exonuclease, subjected to shotgun sequencing, assembled, and compared to pMAR2 of tEPEC-E2348/69 to determine homology within the bundle-forming pilus (BFP) operon. The PER operon was also examined due to its known involvement in the transcriptional regulation of the BFP operon (Figure 4(b)).^33,34^ Isolate CE102 contains the entire BFP operon with high similarity to pMAR2 among all genes (Figure 4(c)). In addition, *perB* and *perC* within the PER operon retain high similarity to pMAR2, however, *perA* is severely truncated with nearly half of the protein missing in isolate CE102 (Figure 4(c)). Isolates CE116 and CE131 are similar in their BFP/PER operons in which both lack the entire PER operon and the majority of the BFP operon genes (Figure 4(c)). Only 3 (*bfpA, bfpG*, and *bfpB*) out of 14 BFP operon genes are present in CE116 and CE131, and *bfpB* is severely truncated (Figure 4(c)). Although isolate CE152 retains 8 out of 14 genes of the BFP operon with high identity to pMAR2, other genes involved in pre-bundlin cleavage and alignment, and the minor pilins are absent (Figure 4(b-c)). Furthermore, the PER operon is extensively altered in isolate CE152 with only *perC* retaining high homology to pMAR2 (Figure 4(c)).

To further understand how alterations in the PER operon contribute to *bfpA* expression, reverse-transcriptase quantitative PCR (RT-qPCR) was used to examine *bfpA* transcript levels at mid- and late-log growth phases for all four *bfpA*-positive isolates (CE102, CE116, CE131, CE152) (Supplemental Figure 3). Comparisons were made to *bfpA* transcript levels in tEPEC-E2348/69 at mid-log growth as BfpA protein levels are known to dramatically increase in mid-log phase and remain stable well into stationary phase.^35^ The *bfpA* transcript levels of all four isolates are significantly lower than those of tEPEC-E2348/69 regardless of growth phase (Figure 4(d)). Increases in *bfpA* transcript levels are detected during late-log phase compared to mid-log phase for all four *bfpA*-positive isolates, however, the increase is only significant for CE102 (Figure 4(d)). CE102 transcript levels in late-log phase are significantly higher than those of the other *bfpA*-positive strains (CE116, CE131, and CE152) at mid- and late-log phase (Figure 4(d)). These data indicate that BfpA expression is severely compromised in all four *bfpA*-positive isolates compared to tEPEC-E2348/69 likely due to genetic alterations in the BFP and PER operons, however, with longer growth times isolate CE102 is capable of producing higher levels of *bfpA* mRNA.

### Majority of aEPEC clinical isolates display diffuse adherence pattern

**Figure 5.**
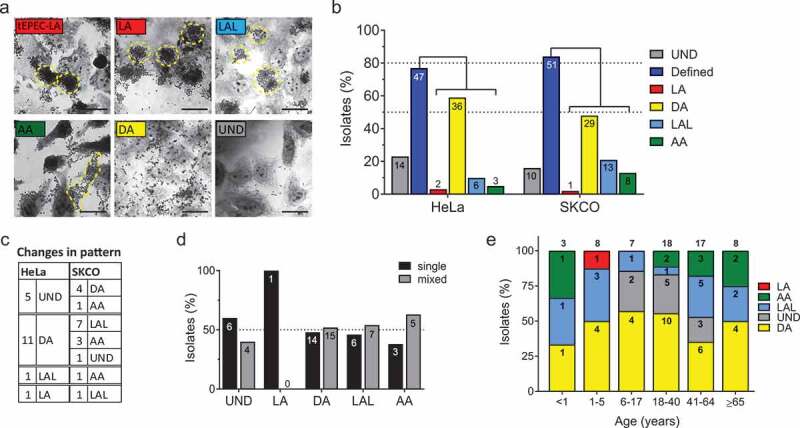
Clinical EPEC isolates display diverse adherence patterns. (a) Representative images of Giemsa stained HeLa epithelial cells infected with isolates for 5 h, exhibiting all described adherence patterns. Yellow dashes encircle clusters of attached bacteria. Localized adherence (LA); localized adherence-like (LAL); aggregative adherence (AA); diffuse adherence (DA); undefined (UND). Scale bar = 10 µm. (b) Percentage of isolates exhibiting each adherence pattern when infecting HeLa or SKCO-15 epithelial cells with “n” displayed for each. More isolates display a defined pattern on SKCO-15 than on HeLa cells. (c) Breakdown of the number of isolates whose adherence pattern originally determined on HeLa cells changed after infection of SKCO-15 cells. Overall, isolates exhibit more robust attachment to SKCO-15 compared to HeLa cells. (d) Similar occurrence of single or mixed EPEC infection status occurs among isolates grouped by adherence pattern. Percentage of isolates for single or mixed infection for each adherence pattern with “n” displayed for each. (e) Adherence pattern proportions for isolates grouped by patient age with “n” displayed within each pattern and total “n” displayed on top of each age-group bar. DA predominates in most age groups and UND is not found in those ≤5 or ≥65 yrs

Previous studies have characterized adherence patterns of EPEC isolates associated with diarrheal outbreaks and have historically used HEP-2 or HeLa epithelial cells originating from human laryngeal and cervical carcinoma, respectively.^36^ We compared attachment to HeLa cells and human colonic epithelial cells, SKCO-15, in order to assess the adherence pattern of clinical EPEC isolates on cells that more closely model the natural target cell of EPEC. Giemsa stain and light microscopy were used to classify adherence patterns as previously described: localized adherence (LA) – large, 3D circular clusters exemplified by tEPEC-E2348/69; localized adherence-like (LAL) – loose 2D circular clusters; aggregative adherence (AA) – large 2D amorphous clusters of bacteria in a “stacked-brick” arrangement; diffuse adherence (DA) – bacteria attached in a randomly scattered manner; undefined (UND) – sparse, individual adherent bacteria with no discernible pattern. The clinical EPEC isolates exhibit all described adherence patterns as represented in Figure 5(a). Most strains display one of the defined adherence patterns (LA, LAL, DA or AA) on both HeLa (77.1%) and SKCO-15 (83.6%) (Figure 5(b)). Only 23.0 and 16.4%, respectively, adhere with an UND pattern (Figure 5(b)). DA predominates on both cell lines, while only 1–2 strains exhibit LA (Figure 5(b)). 29.5% (18/61) of clinical EPEC isolates display different adherence patterns on HeLa and SKCO-15 cells (Figure 5(c)). Of those that differ between cell lines, the majority (88.8%) display a more robust adherence pattern (LAL or AA) on SKCO-15 as opposed to UND or DA on HeLa cells (Figure 5(c)). Of the 4 *bfpA*-positive strains, only one (CE102) displays LA on both HeLa and SKCO-15 cells confirming the important role BfpA expression plays on adherence pattern.

Next, we questioned if specific patient demographics correlate with adherence pattern. Adherence pattern displayed on SKCO-15 cells is not predicted by co-infection status as each adherence pattern is equally represented by EPEC from samples positive for single and multiple pathogens (Figure 5(d)). Adherence pattern is also not associated with a specific age group, however, UND was not identified among isolates from infants and young children (0–5 yrs) nor was it identified in samples from elderly adults (≥65 yrs) (Figure 5(e)). DA is the predominate adherence pattern in most age groups. Interestingly, the LAL adherence pattern is more prevalent than the UND pattern in those 41–60 years of age compared to those 18–40 years old (Figure 5(e)). In summary, clinical EPEC strains are more adherent to colonic cells than to HeLa cells, display diverse adherence patterns across all age groups with DA predominating, and co-infection status does not predict adherence pattern. Based on adherence pattern, the majority of clinical EPEC isolates functionally represent aEPEC strains.

### Clinical EPEC isolates with defined adherence pattern attach at higher levels

**Figure 6.**
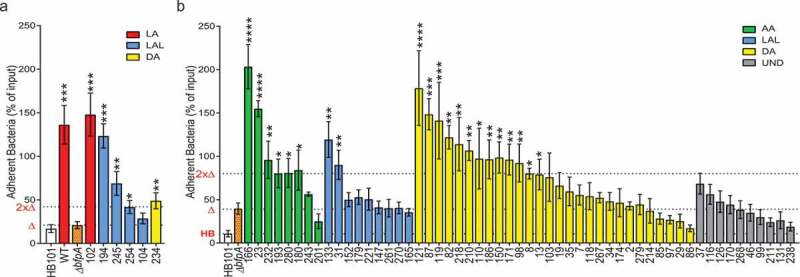
Isolates exhibiting a defined adherence pattern attach to host cells at higher levels than tEPEC lacking *bfpA*. (a-b) SKCO-15 monolayers were infected with indicated clinical EPEC isolate for (a) 2.5 or (b) 5 h, washed, and attached bacteria enumerated and compared to attachment of tEPEC lacking *bfpA* (Δ*bfpA*). HB101 and tEPEC-E2348/69 were used as negative and positive controls, respectively. The attachment level of HB101 (HB), Δ*bfpA* (Δ), and 2 times the level of Δ*bfpA* (2xΔ) are indicated along the y-axis. Mean ± SEM is plotted for each of 3 biological replicates performed at least in duplicate. (a) Six isolates cause cells to lift at 5 h post-infection; therefore, attachment to host cells was assessed for these at 2.5 h. 5/6 clinical isolates tested at 2.5 h have significantly higher levels of attachment compared to Δ*bfpA* regardless of adherence pattern, while one attaches at a similar level to Δ*bfpA* and HB101. (b) The majority of AA and nearly half of DA isolates attach at higher levels than Δ*bfpA*, while attachment of only 2 LAL are significantly higher than Δ*bfpA*. None of the UND isolates attach at significantly higher levels than Δ*bfpA*. 1-way ANOVA was performed with LSD Fisher post-hoc tests; * *p* < .05, ** *p* < .01, *** *p* < .001, **** *p* < .0001 compared to Δ*bfpA.*

To further characterize the adherence properties of the 61 clinical EPEC isolates, attachment levels were assessed at 2.5 and 5 h post-infection on SKCO-15 cells. HB101, a nonpathogenic strain of *E. coli*, and tEPEC-E2348/69 were included as negative and positive controls, respectively. As the majority of isolates are aEPEC, Δ*bfpA* was used as a comparator for baseline attachment to host cells. Most strains were analyzed at 5 h post-infection, however, a few clinical EPEC isolates as well as tEPEC-E2348/69 cause cells to lift at 5 h, therefore, attachment levels were determined only at 2.5 h post-infection (Figure 6(a)). At 2.5 h post-infection, all but one clinical EPEC isolates attach to SKCO-15 cells at higher levels than Δ*bfpA*, regardless of adherence pattern (Figure 6(a)). As anticipated, strain CE102, which harbors *bfpA* and displays LA, has attachment levels similar to tEPEC-E2348/69.

At 5 h post-infection, the majority (6/8) of clinical EPEC isolates displaying AA and nearly half (13/29) of DA strains attach at levels higher than Δ*bfpA* (Figure 6(b)). In contrast, only 2 of 9 LAL strains examined at 5 h and none of the UND strains have levels of attachment greater than that of Δ*bfpA* (Figure 6(b)). Together, these data indicate that the majority of clinical EPEC isolates with a defined adherence pattern have enhanced attachment properties compared to Δ*bfpA*, however, most demonstrate less attachment than tEPEC.

### Majority of clinical EPEC isolates form actin-rich pedestals

**Figure 7.**
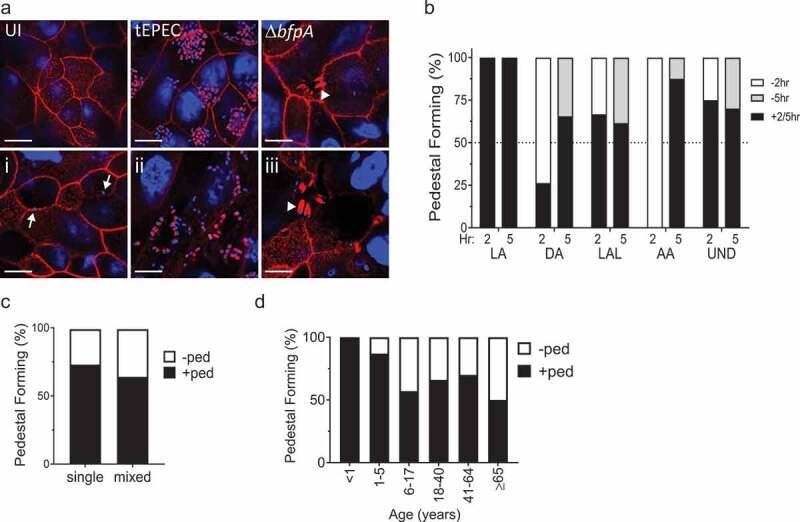
Majority of clinical EPEC isolates form actin-rich pedestals. (a) SKCO-15 monolayers were infected with clinical EPEC isolates for 2–5 h and stained for F-actin with BODIPY-Phalloidin (red); bacterial and host cell nuclei were stained with Hoescht (blue). Representative images reveal F-actin at cell-cell contacts and within the brush border in uninfected (UI) cells. Cells infected with tEPEC-E2348/69 exemplify large dense pedestals associated with microcolonies. tEPEC-E2348/69 lacking *bfpA* (Δ*bfpA)* induce elongated pedestals beneath individually attached bacteria. *(i)* Some isolates do not form pedestals although attached bacteria are apparent (white arrow), (*ii)* while others form robust pedestals, and (*iii)* some form elongated pedestals similar to Δ*bfpA* (white arrowhead). In total, 68.9% (42/61) of clinical EPEC isolates form pedestals by 5hr post-infection. Scale bar = 10 µm. (b) Proportion of pedestal-forming isolates at 2 or 5 h post-infection grouped by adherence pattern. An equal proportion of isolates from LA, LAL, and UND patterns form pedestals at 2 hrs compared to 5hrs. In contrast, EPEC displaying DA and AA patterns require longer times for pedestal formation. (c) Similar proportions of isolates from EPEC sole infection (single) and those from a mixed infection form pedestals. (d) Proportion of pedestal-positive isolates grouped by patient age. Although not significantly different, the majority of isolates from patients ≤5 years old form pedestals, however, the proportion of pedestal forming isolates from those ≥6 years old declines to <50%

Pedestal formation, defined as actin-rich clusters beneath intimately attached bacteria, is a well-characterized virulence phenotype of tEPEC.^37^ Therefore, pedestal formation by clinical EPEC strains was assessed 2 or 5 h post-infection of SKCO-15 monolayers. As expected, F-actin is seen in uninfected cells at the brush border and at cell-cell contacts (Figure 7(a)). tEPEC-E2348/69 induces dense clusters of F-actin under attached bacterial microcolonies (Figure 7(a)).^38^ In contrast, Δ*bfpA* induces pedestals often associated with long actin tails under single-attached bacteria (Figure 7(a)).^39^ Some isolates attach without evidence of pedestal formation (Figure 7(a-i)), while others robustly form pedestals (Figure 7(a-ii)) and several form pedestals similar to infection with Δ*bfpA* (Figure 7(a-iii). In total, 69% (42/61) of clinical EPEC isolates form pedestals. Interestingly, some isolates, typically those displaying LA, LAL, and UND patterns, form pedestals after only 2 h of infection (Figure 7(b)). At 5 h post-infection, >50% of clinical EPEC isolates form pedestals regardless of adherence pattern (Figure 7(b)). There is no significant difference in pedestal formation by EPEC isolates from single (22/61) versus mixed (20/61) infections (Figure 7(c)). Although not significantly different, 91% (10/11) of clinical EPEC isolates from patients aged ≤5 years formed pedestals, while a smaller number of isolates, 64% (32/50), from patients >5 years old display this phenotype (Figure 7(d)). Analysis of more EPEC clinical isolates is needed to determine if this trend is significant. Overall, the majority of clinical EPEC isolates in this study form actin-rich pedestals regardless of adherence pattern, co-infection status, or patient age.

### Barrier disruption by clinical EPEC isolates

**Figure 8.**
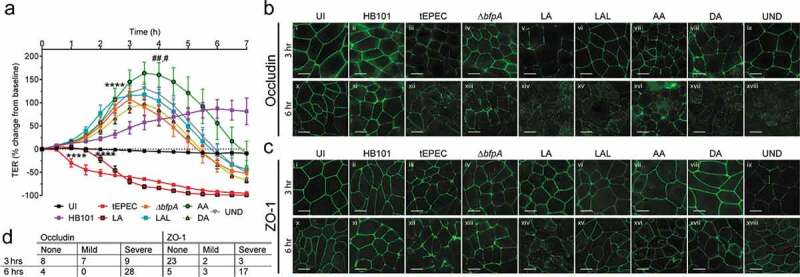
Adherence pattern of clinical EPEC isolates does not influence the impact on barrier function of intestinal epithelial monolayers. (a-d) SKCO-15 monolayers grown on Transwells were infected with a sub-set of clinical EPEC isolates exhibiting different adherence patterns, tEPEC-E2348/69, tEPEC lacking *bfpA* (Δ*bfpA*), and nonpathogenic HB101 or left uninfected (UI). (a) Transepithelial electrical resistance (TER) was measured every 30 minutes post-infection and is displayed as percent change from baseline. All but one isolate initially increase but ultimately decrease TER with kinetics similarly to Δ*bfpA*. One *bfpA-*positive isolate that attaches with LA, decreases TER similarly to tEPEC, although the drop is delayed. 2-way ANOVA with Tukey post-hoc test was performed and mean ± SEM are plotted. Significant differences in the increase in TER are seen between AA, DA, LAL, UND isolates and Δ*bfpA* compared to UI at 3 h, and between tEPEC at 1 hour and LA at 2 h compared to UI (**** *p* < .0001). LA isolate remains statistically higher than tEPEC at 2 hrs post infection (**** *p* < .0001). TER of monolayers infected with isolates displaying AA is significantly higher than those infected with Δ*bfpA* and DA isolates at 4 h (##p < .01, #p < .05). (b and c) Immunofluorescence microscopy of occludin and ZO-1 at 3 (i–ix) or 6 h (x–xviii) post-infection. Scale bar = 10 µm. Occludin and ZO-1 disruption is categorized as ‘mild’ if a cytoplasmic increase or reduction at cell-cell contacts is seen as in (c-iii) and as ‘severe’ if a marked loss from cell-cell contacts leading to a “beads-on-a-string” appearance and/or extensive cytoplasmic accumulation is observed as in (b-xii). (b) Occludin is unperturbed or only mildly disrupted by HB101, Δ*bfpA*, and isolates with LAL, AA, DA, and UND pattern at 3 hrs post-infection but is severely disrupted by tEPEC and an LA displaying isolate at this time point. All strains, except HB101, severely disrupt occludin by 6 hrs post-infection. (c) tEPEC and one isolate exhibiting LA mildly and severely disrupt ZO-1 at 3 and 6 hrs post-infection, respectively, while Δ*bfpA* and isolates with all other adherence patterns require longer infection times to do so. HB101 does not disrupt ZO-1 at either time-point. (d) Summary of the number of isolates perturbing each TJ protein at both timepoints

Barrier disruption is a well-described virulence-phenotype of tEPEC which contributes to diarrhea.^40^ tEPEC-E2348/69 decreases the transepithelial electrical resistance (TER) of intestinal epithelial monolayers characterized by an initial drop of approximately 40% 1 h post-infection with a continual decline as infection progresses (Figure (8a)).^29–31^ In contrast, Δ*bfpA* causes an initial rise in TER, peaking at 3.5 h post-infection, followed by a precipitous drop over the next 4 h (Figure (8a)).^3^ To ensure this effect was not the result of bacterial overgrowth, SKCO-15 monolayers were infected with nonpathogenic HB101 and TER was followed. A steady rise in TER was observed over the course of infection with HB101 (Figure 8(a)). 96% (27/28) of isolates exhibit TER kinetics similar to those of Δ*bfpA*, inducing a significant initial rise which peaks at 3–4 h post-infection then subsequently drops as the infection progresses (Figure 8(a)). The initial rise in TER by strains displaying AA pattern was significantly higher than that induced by Δ*bfpA* and those exhibiting DA pattern (Figure 8(a)). Isolate CE102, which is *bfpA-*positive, exhibits LA, and induces robust pedestal formation, is the only strain that significantly decreases TER by 2 h post-infection (Figure (8(a)). This decrease, however, is delayed compared to tEPEC-E2348/69 (Figure 8(a)).

To further investigate the impact of clinical EPEC isolates on TJ, occludin and ZO-1 were analyzed by immunofluorescence microscopy at 3 and 6 h post-infection (Figure 8(b-c)). The normal distribution of occludin and ZO-1, localized to TJ along the cell-cell contacts, is shown in uninfected (UI) cells (Figure 8(b-i)(c-i)). Disruption is categorized as ‘mild’ if either occludin or ZO-1 are increased in the cytoplasm or arereduced at areas of cell-cell contact (Figure 8(c-iii)). Disruption is characterized as ‘severe’ if there is marked loss from cell–cell contacts leading to a “beads-on-a-string” appearance, extensive accumulation in the cytoplasm, or both as exhibited by tEPEC (Figure 8(b-xii)). Infection with HB101 for 3 h does not alter the localization of occludin or ZO-1 compared to UI cells (Figure 8(b-ii)(c-ii)). In contrast, at 3 h post-infection, tEPEC-E2348/69 severely disrupts occludin and modestly alters ZO-1 (Figure 8(b-iii)(c-iii)). Infection with Δ*bfpA* only mildly disrupts occludin and does not disrupt ZO-1 at 3 h post-infection (Figure 8(b-iv)(c-iv)). CE102, the strain, which exhibits LA on SKCO-15 cells and drops TER early in the course of infection, causes severe and mild disruption of occludin and ZO-1, respectively, at 3 h post-infection (Figure 8(b-v)(c-v)). All other clinical isolates induce either mild (Figure 8b-vii)) or no disruption of occludin or ZO-1 at this timepoint (Figure 8(b-vi/ix)(c-vi-viii)). In contrast, nearly all clinical EPEC strains severely disrupt both occludin and ZO-1 by 6 h post-infection (Figure 8(b-xiv-xviii)(c-xiv-xviii)), as does Δ*bfpA* (Figure 8(b-xiii)(c-xiii)). After 6 h, uninfected cells remain unperturbed (Figure 8(b-x)(c-x)), those infected with HB101 show very mild or no disruption of occludin or ZO-1 (Figure 8(b-xi)(c-xi)), and as expected, tEPEC-E2348/69 infection induces more severe effects (Figure 8(b-xii)(c-xii)). As summarized in Figure 8(d), all of the tested EPEC isolates cause severe disruption of occludin at 6 h post-infection, showing marked loss from TJs and mis-localization to the cytoplasm. 81% (17/21) of strains induce severe ZO-1 disruption at 6 h post-infection. These data indicate that occludin is universally disrupted by all clinical EPEC isolates examined and the effects on ZO-1 are also substantial. Adherence pattern does not predict the severity of TJ disruption.

## Discussion

The majority of the clinical EPEC isolates in this study represent atypical EPEC as determined by the lack of *bfpA*. The high prevalence of aEPEC isolates is in accordance with recent reports on the predominance of aEPEC in developed and developing nations alike.^13,24,41^ However, the high global prevalence of aEPEC in symptomatic and in some asymptomatic individuals is perplexing and the pathogenic mechanisms of aEPEC remain unclear. We found that EPEC were equally distributed between samples identifying only EPEC and as part of co-infections. However, relative EPEC load was more frequently greater in samples with mixed infections. Phenotypic analysis of 61 clinical EPEC isolates in our study reveals that all but one of these strains have either diminished *bfpA* transcript levels or are completely devoid of *bfpA* and thus should be considered aEPEC. Despite this, most isolates examined in this study induce physiological effects known to contribute to diarrhea caused by tEPEC, including high levels of bacterial adherence, cytoskeletal rearrangement into pedestals, and disruption of TJ structure and function. These data reinforce aEPEC as an emerging phenotypically diverse global pathogen.

EPEC prevalence in our study was similar to that reported from other developed nations. EPEC was the most frequently detected pathogen by GIP in a European multicenter evaluation, present in 27.9% of samples, and a multicenter US evaluation of GIP reported EPEC was present in 22.4% of samples.^12,13^ Interestingly, data from LUMC show prevalence to be significantly greater during summer months, a trend that has been noted by others for diarrheagenic *E. coli* but not specifically for EPEC.^13,42,43^

To begin to understand the significance of high EPEC prevalence, we questioned if EPEC bacterial loads differ among diarrheal samples as previous reports indicate that high EPEC load in stool samples is associated with symptomatic status.^27^ GIP is a very sensitive assay, achieving a reported 100% sensitivity with only 200 input bacteria.^32^ Using genomic DNA from a sample of tEPEC-E2348/69 culture and qPCR, we detected 16S rDNA and *eaeA* efficiently with similar detection limits as GIP. Surprisingly, nearly half of the GIP-EPEC-positive stool samples contained levels of *eaeA* below the limit of detection of the qPCR assay. However, this was likely due to limitations in the quality and quantity of genomic DNA extracted from diarrheal stool samples owing to its complexity. Such a low detection threshold by GIP raises the question of the value of this level of sensitivity for EPEC infection. In human volunteer studies, ingestion of ~10^10^ CFU of EPEC, whether typical or atypical, was required to induce diarrhea.^14^ Furthermore, in the case of aEPEC and Δ*bfpA, eaeA*-positive stool cultures were detected even in individuals without diarrhea.^15,16^ Combined with the prevalence of EPEC detected in food and animal sources, it is plausible that GIP could be identifying ingested EPEC passing through the digestive tract without establishing colonization.^44–49^ In addition, since detection is based solely on PCR, even non-viable organisms would be detected.

Despite the high number of samples with EPEC load below the limit of detection, we questioned if EPEC load differed between samples containing EPEC as the sole pathogen versus EPEC present with other pathogens. We found that samples from patients with co-infecting enteric pathogens more frequently contained higher EPEC loads. Our data suggest that EPEC infection may promote the growth of other enteric pathogens, or alternately, that EPEC could flourish and become pathogenic in the setting of other pathogens, host inflammation, or dysbiosis induced by an underlying condition.^50–52^ This hypothesis is in agreement with a previous study suggesting that artificially introduced nonpathogenic *E. coli* grow to significantly greater abundance in mice under conditions of dextran sodium sulfate-induced colitis or inflammation-prone IL-10 knockout mice.^53^ In humans, a volume of evidence suggests that blooms of Enterobacteriaceae can occur in the context of gut inflammation and have been associated with pathogenic manifestations such as Crohn’s disease, ulcerative colitis, enhanced susceptibility to *Salmonella enterica* serovar typhimurium or *C. difficile* infection, and even colorectal cancer.^54–59^ In addition, the recent report that EPEC unknowingly present in fecal microbiota transplant products derived from healthy donors can cause disease in those with recurrent *C. difficile* supports the contention that host status is an important factor in pathogenesis.^54^ Future studies are warranted to correlate diarrheal severity with bacterial load and to compare bacterial load in symptomatic versus asymptomatic individuals. In addition, a better understanding of the contribution of host factors to aEPEC pathogenesis is needed.

Using standard culturing techniques and colony PCR, we were able to purify 61 *eaeA*-positive isolates from a total of 277 samples with an isolation rate of 22%, which is similar to other reports.^55^ Patient demographic data, *bfpA* detection, and virulence-associated phenotypes were analyzed for each of the 61 EPEC isolates as summarized in Supplementary Table 2. Clinical EPEC isolates were equally distributed between males and females as well as between samples with single and mixed infections. EPEC detection spanned all age groups ranging from <1-102 years old, however, the majority of isolates obtained were from adults 18–64 years old. Further analysis is needed to determine the prevalence of EPEC among children and adults in the US.

Typical or atypical EPEC designation is determined by the detection of *bfpA* using one of two different methods: first, by PCR using single or multiplexed reactions for *bfpA*, or second, by using a probe spanning a region over the BFP operon.^60–62^ Probe technology is difficult in most clinical settings and the majority of EPEC analyses do not include the typical or atypical EPEC designation by PCR. In addition, the PER operon or other transcriptional regulators known to affect BFP expression are often not, if ever, examined. In the present study, colony PCR screens for *bfpA* reveal that the majority of isolates are aEPEC and only 4 isolates are *bfpA*-positive and thus could be deemed tEPEC. However, upon further genetic and transcriptional analysis, one can assume that BfpA protein levels are severely compromised in all but one isolate, CE102, due to the lack of *bfpA* or lack of *bfpA* transcript which likely resulted from mutations in the PER operon. Even if BfpA was expressed in the remaining *bfpA*-positive isolates (CE116, CE131, and CE152), genetic analysis reveals severe truncations and alterations in the BFP operon likely rendering it nonfunctional in these strains. A complex interplay between the proteins of the BFP operon are needed for bundling processing, secretion, and pili formation and retraction.^35,63,64^

The entire BFP operon is present in isolate CE102, however, only low but detectable levels of *bfpA* transcript were present potentially due to the *perA* truncation. Despite this, isolate CE102 retains all virulence-associated phenotypes examined, including LA, a high level of attachment, and induction of pedestal formation. CE102 also disrupts TJ structure and function with similar kinetics to tEPEC-E2348/69, however, the decrease in TER is delayed in CE102 compared to tEPEC-E2348/69. This lag may be due to a delay in BfpA expression as *bfpA* transcript is not detected until the late-log phase of growth. Interestingly, CE147 is the only other isolate to attach in a LA pattern on HeLa cells despite being *bfpA*-negative. However, on the intestinal cell line SKCO-15, CE147 exhibits a LAL pattern, attaches at similar levels to *ΔbfpA*, and disrupts TJ function and structure similar to other aEPEC strains while retaining the ability to form pedestals. Thus, CE102 is the only clinical isolate that should be considered tEPEC with the remainder of the isolates being aEPEC based on their lack of *bfpA* transcript, incomplete BFP operon, and lack of LA on intestinal cells. Since determination of BFP presence or functionality is difficult in a clinical setting, further studies are needed to determine the significance of variations in both genetic and functional differences of the BFP and PER operons of aEPEC isolates to improve the paradigm of atypical designation for clinical EPEC isolates.

Most clinical aEPEC isolates from this study exhibited a DA pattern, although all aEPEC adherence patterns were represented (LAL, AA, DA, UND). Diffuse adherence was originally attributed to the locus for diffuse adherence (*lda*).^56^ However, this locus is not found in every aEPEC strain and a variety of other adhesins likely contribute to the various adherence patterns of aEPEC.^25^ In addition, diffuse adherence is displayed by strains found in both symptomatic and asymptomatic matched controls, adding controversy to their association with diarrheal disease.^36,57,58^ More isolates displayed a defined adherence pattern on intestinal SKCO-15 cells versus cervical HeLa cells. Our study showed no clear association between any adherence pattern and sole or co-infection status. Patient age also did not correlate with specific adherence patterns. However, undefined adherence was not found in those aged <5 and **≥**65 years; the significance of this is yet to be determined.

Adherence pattern and bacterial attachment level were not correlated in this study, suggesting different mechanisms of interaction between host-bacteria and bacteria-bacteria as was suggested for the EAEC.^59^ However, most of the isolates with a defined adherence pattern had higher levels of attachment than Δ*bfpA*. Similarly, a high proportion (68.9%) of pedestal-forming aEPEC isolates was identified in this study and was slightly higher than reported in other recent studies showing pedestal formation in 58.5% of isolates.^55^ This difference may be accounted for by the use of intestinal epithelial cells in the current study, as opposed to HeLa. Therefore, the majority of clinical aEPEC isolates retain the ability to form A/E lesions, a characteristic hallmark of tEPEC pathogenesis.^11^

In addition to A/E lesions, perturbation of ion secretion/absorption and disruption of barrier function contribute to tEPEC-induced diarrhea.^65^ All aEPEC isolates from this study alter barrier function of intestinal epithelial monolayers *in vitro* as shown by an initial increase and subsequent decrease in TER as occurs with Δ*bfpA* infection.^59^ It is hypothesized that the increase in TER is caused by tightening of TJ in response to bacteria lacking a T3SS or deficient in colonization, and is counteracted by TJ-disrupting effectors secreted by wild-type tEPEC.^59^ Also, aEPEC isolates in this study show universal disruption of occludin localization and nearly all disrupt ZO-1 localization at infection times that correspond to decreased TER, a known virulence phenotype of tEPEC.^40^ We hypothesize that the initial rise in TER induced by aEPEC infection is due to the lack of BFP retraction and consequent delay in translocation of T3SS effectors into host cells.^3^ Once sufficient levels of T3SS effectors are translocated into host cells, alterations in TJ structure and function ensue. In addition, the lack of the PER operon in aEPEC strains could delay effects on TJ structure and function as PerC (of the PER operon) is a known activator of genes on the LEE pathogenicity island.^66,67^

The analysis of *in vitro* virulence phenotypes of aEPEC isolates in the current study indicates that while some characteristic tEPEC pathogenic mechanisms are retained, many isolates have unique characteristics. For instance, pedestal formation was not detected with several isolates despite having robust virulence phenotypes *in vitro*, such as host cell detachment from the culture plate, high levels of attachment to host cells, and defined adherence patterns. These data suggest that some aEPEC isolates have pathogenic mechanisms that differ from tEPEC. Numerous virulence mechanisms or genetic associations with disease have been proposed, such as the non-LEE-encoded T3SS effector cycle-inhibiting factor (Cif) known to induce irreversible G2 arrest in host cells, plasmid-encoded toxin (Pet) which disrupts actin stress fibers and cytoskeletal contractility ultimately leading to detachment, or enterohemolysin encoded by *ehxA*, positively associated with aEPEC from children with diarrheal disease in Norway and Brazil.^68–71^ aEPEC could induce diarrhea by acquiring virulence factors such as toxins, adhesins, or by perturbing the native microbiota thus allowing other pathogens to flourish.^6,21,72^ Thus, in-depth genetic analysis of aEPEC isolates for additional virulence determinants is warranted.

It should be noted that *in vitro* virulence analyses do not necessarily reflect virulence within the host. For instance, clinical aEPEC isolates from serogroup O125:H6 did not trigger pedestal formation on HeLa cells *in vitro*, however, pedestals and A/E lesions were present after aEPEC *ex vivo* infection of human terminal ileum.^73^ In addition, aEPEC isolates may retain a fully functional LEE but be negative for A/E lesions *in vitro*.^74^ Numerous cell types have been historically used to study EPEC infections, including HeLa, HEp-2, Caco-2, T84, TC-7, and more. We examined adherence patterns in the historically used cervical epithelial cell line, HeLa, and found differences in the attachment patterns of certain isolates when human colonic SKCO-15 cells were used. These differing results could be due to the vast genetic variability of aEPEC isolates and may contribute to tropism for different intestinal segments of the host. Indeed, subtypes of intimin show tropism to different host cell types.^75,76^ tEPEC is known to infect the small bowel, while EHEC can infect both the small and large intestine, however, nothing is known about the tropism of various aEPEC isolates for different intestinal segments. Determination of the specific intestinal tropism of aEPEC is imperative as the pathophysiology and severity of diarrhea vary depending on the segment of bowel affected.^77^ Therefore, the study of virulence-associated phenotypes of aEPEC isolates in more physiological relevant cells lines such as colonic SKCO-15, small intestinal-like Caco-2, or ideally in human enteroid/colonoid models is warranted.

In conclusion, the results of this study suggest that aEPEC represents the majority of EPEC strains detected in the US, supporting a growing body of literature proclaiming the heterogeneity of genotype, phenotype, and virulence ascribed to aEPEC. The overall clinical significance of EPEC detected by PCR remains uncertain, yet *in vitro* analysis reveals that most strains are capable of inducing phenotypes associated with virulence. More clear sub-divisions of aEPEC are needed to distinguish nonpathogenic from pathogenic strains. This is especially important as a recent prospective study of cancer patients with diarrhea of suspected infectious etiology found EPEC to be the most detected pathotype of diarrheagenic *E. coli*, present in 35/311 (11.3%) of samples.^43^ Persistent colonization and infection by aEPEC may lead to chronic inflammation or more severe long-term perturbations in the gut. The ability of EPEC to form A/E lesions and alter paracellular flux of intestinal epithelia could potentially play a role in chronic disease states, as well as promote perturbations in microbiome composition that could cascade to more severe symptoms in infected hosts.^43^ The long-term consequences of persistent aEPEC colonization are unknown and warrant further investigation in view of their widespread prevalence, genetic and phenotypic diversity, and the unknown contribution of host susceptibility.

## Materials and methods

### Diarrheal stool samples

Diarrheal stool samples from patients with suspected infectious etiology that tested positive for EPEC by GIP were obtained from the clinical microbiology laboratory at Loyola University Medical Center (LUMC) with IRB exempt status approval for retrospective analysis. The samples stored at 4°C in Cary-Blair transport media were obtained within 3–5 days of GIP analysis. Upon acquisition, stool samples were streaked onto MacConkey II agar plates and incubated aerobically overnight at 37°C, then stored at 4°C for no more than 7 days for further analysis. Additionally, ~20 µL of stool sample was used to inoculate 5 mL of LB broth and incubated aerobically at 37°C with shaking at 250 rpm overnight, and glycerol stocks were made and stored at −80°C. GIP analysis reports were obtained to determine the sole (EPEC only) vs. co-infection (multiple pathogens detected) status of patients, temporal information on infections, and patient demographics.

### Nucleic acid manipulations

Genomic DNA was extracted from stool samples using the QIAamp PowerFecal DNA Kit as described (Qiagen, 12830–50). Genomic and plasmid DNA extracts of tEPEC-E2348/69 and clinical EPEC isolates were prepared from overnight LB cultures using DNeasy Blood and Tissue kit (Qiagen, 69504) or the QIAamp PowerFecal DNA kit and Plasmid kit (Qiagen, 10023), respectively, according to manufacturers’ instructions. 100 µL plasmid preparations in 10 mM Tris-Cl were added to 1 mL EX buffer and further treated with exonuclease according to the Large-Construct kit (Qiagen, 12462) instructions, with volumes adjusted appropriately to be purified on 20 μg Tip-20 columns (Qiagen, 10223). Plasmids preparations were then run on 0.8% agarose gel, stained with ethidium bromide, and visualized using AlphaImager (Alpha Innotech) to assess removal of genomic DNA.

For RNA extraction, tEPEC-E2348/69 and clinical EPEC isolates were sub-cultured at 1:33 from over-night cultures in LB media into bacterial infection media, grown to mid- and late-log phase, bacteria pelleted, and resuspended in 600 μL Nucleoprotect (Takara, 740400.5). RNA was extracted using NucleoSpin RNA kit according to the manufacturer’s instructions except 2 mg/mL of lysozyme was used and incubated with bacterial cells for 30 min (Takara, 740955.5). 1 μg of total RNA input was used to create cDNA using PrimeScript RT Reagent Kit with gDNA Eraser following the manufacturer’s instructions (Takara, RR047A).

### Quantitative PCR

To determine bacterial load, Taqman probe quantitative PCR reactions was employed using AmpliTaq Gold 360 Master Mix (Applied Biosystems, 4398881) and were carried out using previously described primers for *eaeA* (0.5 μM) and a newly designed probe (0.4 μM) (Supplemental Table 1).^31^ The amount of total bacteria in stool samples was quantified as a normalization signal using 16S-rDNA universal primers (0.5 μM) and probe (0.2 μM).^29^ qPCR for each sample was run in triplicate alongside triplicate standard curves ranging from 1.38 × 10^5^–1.38x10^−1^ pg of input tEPEC-E2348/69 genomic DNA using the Mastercycler Realplex^2^ (Eppendorf). The PCR cycle parameters were as follows: 95°C (10 min.), 40 cycles of 95°C (30 sec.), 50°C (30 sec.), 72°C (30 sec.), then terminated 72°C (7 min.). Eppendorf RealPlex software was employed for generation and analysis of standard curves and *eaeA*/universal bacterial quantification.

RT-qPCR of *bfpA* transcripts was accomplished using TB Green Premix Ex Taq II (Takara, RR820A) and *bfpA* primers (0.4 μM) and 16S-rDNA universal primers (0.4 μM) (Supplementary Figure 1). 2 μl neat for *bfpA* amplification and 2 μL (1:50) for universal amplification of cDNA input was used in total volume of 25μL reaction. qPCR for each sample was run in triplicate for at least three biological replicates using the Mastercycler Realplex^2^ (Eppendorf). The qPCR cycle parameters were as follows: 95°C (30 sec), 40 cycles of 95°C (8 sec), 50°C (20 sec), 68°C (30 sec), 60°C (15 sec-read fluorescence), followed by melt curve analysis. Eppendorf RealPlex software was employed for generation of *bfpA*/universal Ct values. *bfpA*-mRNA relative expression levels were calculated using the 2^−ΔCt^ method where ΔC_t_ = C_t_(*bfpA*)-C_t_(universal).^78^

### Colony PCR of Gram-negative bacteria

In order to identify *eaeA*-positive bacteria, single colonies were patched onto MacConkey II agar plates and subjected to colony PCR using AmpliTaqGold PCR master mix (Applied Biosystems, 4398881) and *eaeA*-specific primers at 0.5 µM (Supplemental Table 1).^31^ The PCR cycle parameters were the same as in the qPCR for bacterial load. PCR products were electrophoresed on 1.2% agarose gels, stained with ethidium bromide, and visualized using AlphaImager (Alpha Innotech). Primary *eaeA-*positive patches were re-streaked onto fresh MacConkey II agar and incubated as above. Patches from re-streaked colonies were rescreened for *eaeA* to ensure purity of isolated colonies, then grown overnight in Luria-Bertani (LB) broth (BD, 244620) to prepare glycerol stocks and stored at −80°C. Pure *eaeA-*positive isolates were grown on MacConkey II agar and subjected to colony PCR as above, using 0.5 µM primers specific for a 410 bp amplicon of *bfpA* (Supplementary Table 1), whose presence or absence defines typical vs. atypical EPEC, respectively.^62^

### Shotgun sequencing of bfpA-positive isolates

Purified genomic and exonuclease-treated plasmid preparations were submitted to the Loyola Genomic Facility for library preparation and shotgun sequencing. Briefly, samples were prepared with the Illumina Nextera Flex Library Preparation Kit multiplexed with Nextera indexes. Libraries were pooled and sequenced on four Miseq PE250 runs to yield approximately 100x coverage. Paired sequences were assembled and annotated using the PATRIC Comprehensive genome analysis.^79,80^ Homology comparisons of clinical EPEC isolates to tEPEC-E2348/69 were done using Proteome comparison tool on PATRIC.^79,80^

### Epithelial cell culture

Human SKCO-15 colonic and HeLa cervical epithelial cells were used between passages 30–45 and 3–10, respectively, and grown in a 5% CO_2_ incubator at 37°C in Dulbecco’s Modified Eagle Media (DMEM) (Gibco, 31600034) supplemented with 20.8 mM HEPES (US Biological, H2010), 19.4 mM D-dextrose (Fisher, D16-500), 10% FBS (Gibco, 16140071), and 1% penicillin/streptomycin (Gibco, 15140163). Eighteen hours before bacterial infections, cell culture medium was changed to bacterial infection media.

### Bacterial infections

Primary cultures of clinical EPEC isolates were prepared in 5 mL of LB broth and grown at 37°C with shaking at 250 rpm for ~18 h. Then 150 µL of primary culture was inoculated into 5 mL of bacterial infection media consisting of serum- and antibiotic-free 1:1 (v/v) mixture of DMEM:Ham’s F12 (Gibco, 21700075) containing 0.5% mannose (Sigma, M6020) and grown to mid-log phase to reach ~5x10^8^ CFU/mL as previously described.^81^ Bacteria were centrifuged at 1000 rcf for 10 min, the pellet resuspended in 5 mL fresh 37°C bacterial infection media and added to epithelial cells at a multiplicity of infection (MOI) of ~50:1 for the indicated times.

### Adherence pattern determination of clinical EPEC isolates

HeLa and SKCO-15 epithelial cells were plated on glass coverslips in 24-well plates, grown to ~80-90% confluency, and infected for 2.5 or 5 h. Infected monolayers were washed three times with 1x PBS (Fisher, BP399500), fixed with cold methanol (5 min), stained with Geimsa (Sigma, 1092040500) (20 min) at room temperature, washed, and mounted with Permount (Fisher, SP15-100). Images were acquired using phase-contrast light microscopy on Leica DMI4000 (Metamorph software) and processed using Adobe Photoshop 2020.

### Adherence assay

SKCO-15 epithelial cells were cultured in 12-well plates, grown to 90–100% confluency, and infected for 2.5 or 5 h. Infected monolayers were washed 3 times with 1xPBS, 200 μL of 1% Triton X-100 in PBS added to each well and incubated for 10–20 min at 37°C. Cells/bacteria were removed from well with cell scraper after addition of 300 μL of 1xPBS and vortexed vigorously. Serial dilutions of samples at T = 0 and T = 2.5/5 h on LB plates were incubated over-night at 37°C and bacterial colonies enumerated. CFU at T = 2.5/5h were divided by the CFU at T = 0 h equaling the percent of adherent bacteria. All clinical EPEC isolates were tested with at least three biological replicates with at least two technical replicates each and means with standard error of the mean (SEM) reported.

### Fluorescence staining

Epithelial cells were cultured on glass coverslips in 24-well plates, infected with clinical EPEC isolates, washed with PBS and fixed using either cold methanol (5 min) or 3.7% paraformaldehyde in PBS (20 min), washed with PBS, then permeabilized with 0.1% Triton X-100 (5 min) at room temperature and incubated overnight in blocking solution (Invitrogen, 000–105). Cells were labeled with anti-ZO-1 (Invitrogen 617300) in a 1:100 dilution, mouse anti-occludin (Invitrogen 33–1500) at a 1:100 dilution at 4°C overnight, or 1 U of BODIPY-558/568 Phalloidin (Invitrogen, B3475) diluted into 50 µL of blocking solution for 1 h. Cells were incubated with AlexFluor-488 secondary antibodies (Invitrogen, A11034 or A11029) at 1:250 in Invitrogen blocking solution for 2 h at room temperature, nuclei were stained with Hoecsht 33342 (Invitrogen, H3570) and coverslips mounted using ProLong Gold Antifade reagent (Invitrogen, P36934). Images were acquired using either a Leica DMI4000 (MetaMorph software) fluorescence microscope, or a Leica TCS SPE DMI 4000B (LAS X software) confocal microscope and processed with ImageJ and Adobe Photoshop 2020.

### Transepithelial electrical resistance (TER)

SKCO-15 cells were seeded on Transwells (Corning, 3470) at a density of 300,000–500,000 cells per insert. Resistance was measured during monolayer development by Epithelial Volt/Ohm Meter (EVOM) (World Precision Instruments). Once resistance of ~1.0–1.3 kΩ*cm^2^ was reached for 48 h, Transwells were transferred to CellZScope (Nano Analytics) for automated resistance measurement and medium was changed to bacterial infection media 16–20 h prior to infection. Resistance measurements were taken every 30 min post-infection.

### Statistical analysis

All statistical analyses were performed using Graphpad Prism 8 software. 1- and 2-way ANOVA with Tukey or LSD Fisher post hoc tests were used as indicated. Fisher’s exact test was used for tests of differences between proportions, while the Mann-Whitney U-test was used for differences between ranked data sets. For all statistical tests, *p* < .05 was considered statistically significant.

## Supplementary Material

Supplemental Material
