# Supplementary material for: Quantitative analysis and virulence phenotypes of atypical enteropathogenic Escherichia coli (EPEC) acquired from diarrheal stool samples from a Midwest US hospital
Source: Gut Microbes. 2020 Nov 1;12(1):1824562. doi: 10.1080/19490976.2020.1824562 (PMC7644165; doi:10.1080/19490976.2020.1824562)
Supplement: Supplemental Material [file KGMI_A_1824562_SM3536.docx]

Supplemental Table 1. Primer and Probe Sequences Used in this Study.

| Gene target | Orientation | Primer sequence (5’→3’) | Expected amplicon size (bp) | qPCR probe sequences | Reference |
| --- | --- | --- | --- | --- | --- |
| *eaeA* | F  R | ATGCTTAGTGCTGGTTTAGG  CCTTCATCATTTCGCTTTC | 248 | ACTGGTGAAACTGTTGCCGATCTTTCT* | ^26^ |
| *bfpA* | F  R | GTCTGCGTCTGATTCCAATA  TCAGCAGGAGTAATAGC | 408-414 | N/A | ^65^ |
| Universal Bacteria  16S rDNA | F  R | TCCTACGGGAGGCAGCAG  TGGACTACCAGGGTATCTAATCCTGTT | 466 | CGTATTACGCGGCTGCTGGCAC | ^27^ |

* denotes a sequence developed in this study.

**
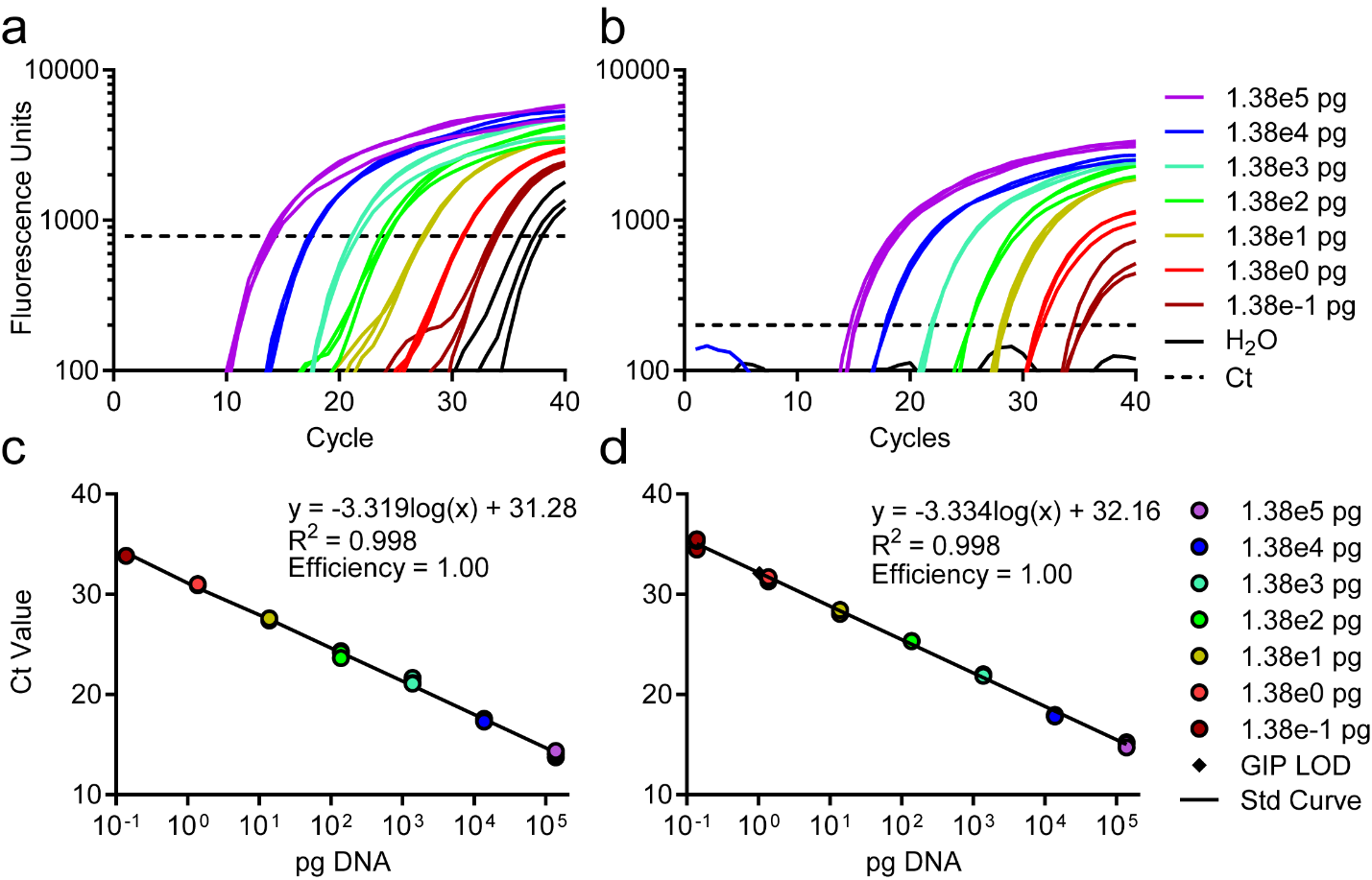
**

**Supplemental Figure 1:** Amplification of *eaeA* and universal bacterial 16S rDNA using serial dilutions of genomic EPEC DNA as template produces standard curves for quantification of EPEC and total bacteria. (a-b) Representative amplification curves measuring fluorescence of serial dilutions of EPEC genomic DNA probing (a) universal bacteria and (b) *eaeA*. Corresponding standard curves were constructed for (c) universal bacteria and (d) *eaeA*. The GIP limit of detection is included in standard curves for reference (200 bacteria or ~1pg DNA, assuming 5.44 fg DNA/bacterium). Standard curves for *eaeA* and universal bacterial rDNA ranging from 0.138-1.38 x 10^5^ pg DNA were successfully generated with 100% amplification efficiency, which are sufficient to detect bacterial quantities below GIP’s stated LOD for EPEC.

**
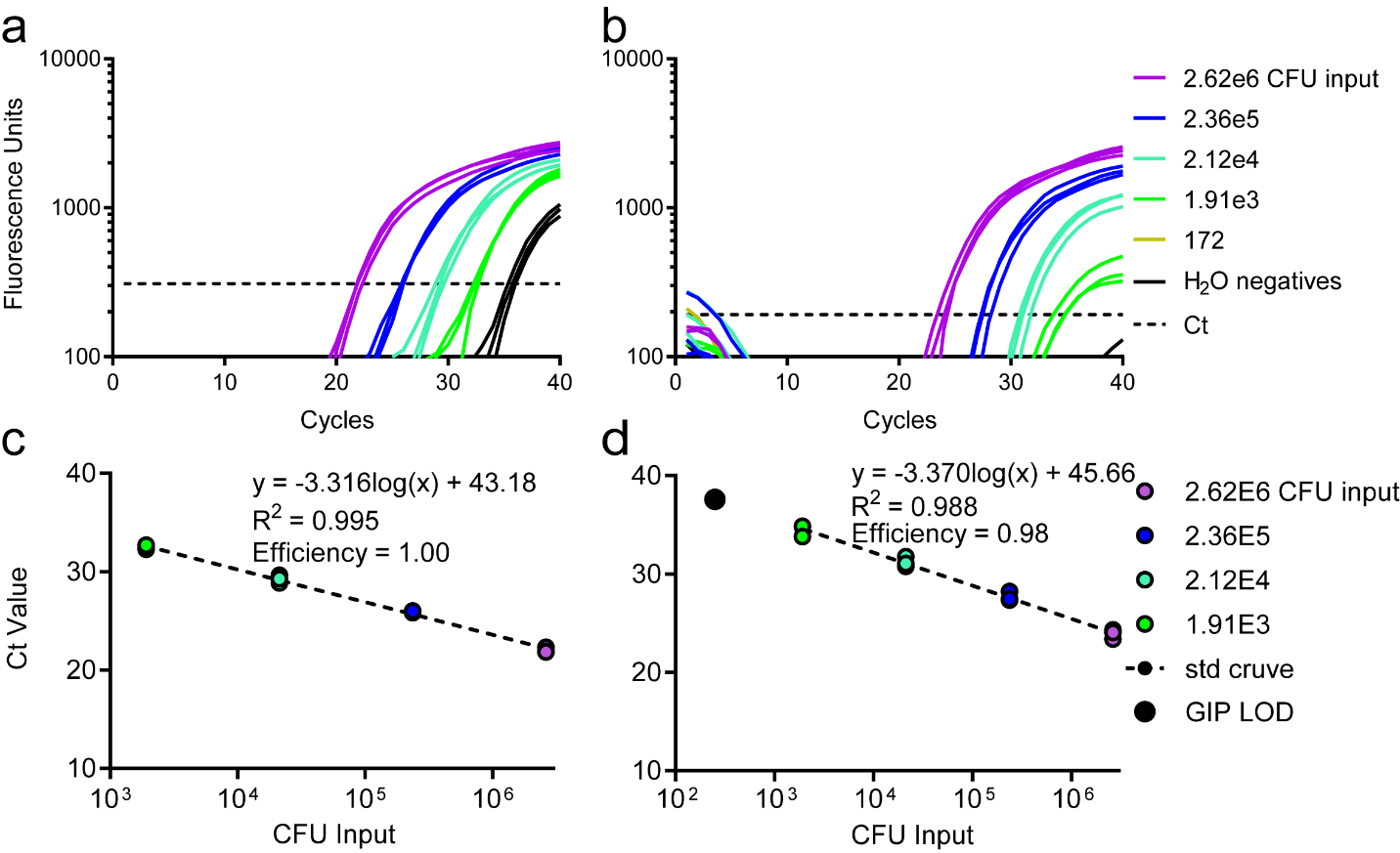
Supplemental Figure 2**: DNA extraction from samples limits the sensitivity of the qPCR assay. (a-b) Amplification curves of serial dilutions of LB cultured tEPEC-E2348/69 in PBS that were extracted using the Qiagen PowerFecal kit as described, probing for (a) universal bacteria and (b) *eaeA*. (c-d) Standard curves were constructed for (c) universal bacteria and (d) *eaeA*. Culture density was quantified by plating the same serial dilutions onto LB agar and enumerating CFUs. Each line represents the resulting *eae* amplification curve of DNA extracts from dilutions containing the color-indicated CFUs. GIP’s limit of detection is included in standard curves for reference (200 bacteria or ~1pg DNA, assuming 5.44 fg DNA/bacterium). No amplification signal was detected in samples containing fewer than 1.91E3 bacteria for either universal or *eaeA* probes, indicating that DNA extraction was a limiting step in the assay. Amplification of the H_2_O negative when probing universal bacteria was most likely the result of bacterial DNA contaminants present in the *taq* polymerase used.

**Supplemental Figure 3**: Growth curves of tEPEC-E2348/69 and *bfpA*-positive clinical EPEC isolates. Over-night LB cultures were sub-cultured into bacterial growth media at 1:33, grown aerobically with shaking, and optical density (OD) measured at 600 nm every 30-60 minutes. Mean±SEM are displayed with mid- and late-log phase indicated for clinical EPEC isolates.
